# Multi-center study of use of the Exeter stem in Japan: a 10-year follow-up report

**DOI:** 10.1007/s00590-024-04001-w

**Published:** 2024-06-01

**Authors:** Tatsuro Sakurai, Hiroshi Fujita, Toshiki Iwase, Kan Sasaki, Naoyuki Katayama, Hiromi Otsuka

**Affiliations:** 1 Department of Orthopedic Surgery, Nagareyama Central Hospital, 2-243-3 Higashihatsuishi, Nagareyama-shi, Chiba 270-0114 Japan; 2 Center for Hip Arthroplasty, Senshunkai Hospital, Nagaokakyo-shi, Kyoto 617-0826 Japan; 3 Department of Orthopedic Surgery, Hamamatsu Medical Center, Hamamatsu, Shizuoka 432-8580 Japan; 4 Department of Orthopedic Surgery, Yamagata Saisei Hospital, Okimachi, Yamagata 990-8545 Japan; 5 Department of Orthopedic Surgery, Hokkaido Orthopedic Memorial Hospital, Sapporo, Hokkaido 062-0937 Japan; 6 Department of Orthopedic Surgery, School of Medicine, Aichi Medical University, Nagakute-shi, Aichi 480-1195 Japan

**Keywords:** Bone cements, Multicenter study, Survival analysis, Total hip arthroplasty

## Abstract

**Purpose:**

Since the introduction of the Exeter stem for clinical use in Japan in 1996, the number of stems used has continued to rise owing to its favorable results. We investigated the outcomes of patients who had previously undergone total hip arthroplasty with the Exeter stem in Japan with a 10-year + follow-up period.

**Methods:**

This retrospective cohort study used clinical and radiographic data of 682 cases of primary total hip arthroplasty performed using the Exeter stem.

**Results:**

The mean postoperative follow-up period was 13.3 years. Femoral-side revision was required in 14 hips, with no cases of aseptic stem loosening-associated revision observed. Kaplan–Meier survival analysis predicted 97.3% 15-year survival when revision for any reason was used as the endpoint.

**Conclusion:**

The obtained findings suggested the excellent long-term stability of the Exeter stem for primary total hip arthroplasty in Japan.

## Introduction

The Exeter stem (Stryker Orthopedics, Mahwah, NJ, USA), a polished, double-tapered, colorless stem, has been delivering favorable postoperative clinical outcomes since its advent in 1969 [1]. Over the years, the Exeter stem has undergone various modifications in terms of materials and surface processing. In 1988, the universal taper design was introduced for clinical use. In 2001, the trunnion shape was changed to a V40 taper. No other modifications have been made to its overall shape; however, the size has been adjusted over time. In 1997, small stems with 30-mm and 33-mm offsets were introduced for use in Asia–Pacific populations. In 1988, stems with 37.5-mm and 35.5-mm offsets were introduced for patients with acetabulum dysplasia or a small build. In 2014, shorter stems of 125 mm with 37.5-mm and 44-mm offsets were introduced for narrow medullary canals and highly curved femurs.

Clinical adoption of Exeter stems has been widespread in Japan since 1996, with their annual usage steadily increasing, establishing them as the preferred choice for cement stems. However, studies involving small Exeter stems with small offsets in Asian populations remain limited. In 2012, Fujita et al. [2] conducted a multi-center study involving 1000 joints with multiple surgeons who received direct surgical training from the hospital facility where the Exeter stem was developed. Interestingly, they reported excellent short-term radiographic results with a mean follow-up period of 4 years. Afterward, as some cases of revision or changes in the area around the femur were observed over time, a long-term follow-up study was conducted. Therefore, in this study, we aimed to investigate the radiographic data of patients who underwent primary total hip arthroplasty (THA) using the Exeter stem in four teaching hospitals in Japan over a mean follow-up period of 13.3 years.

## Methods

### Patients

**Fig. 1:**
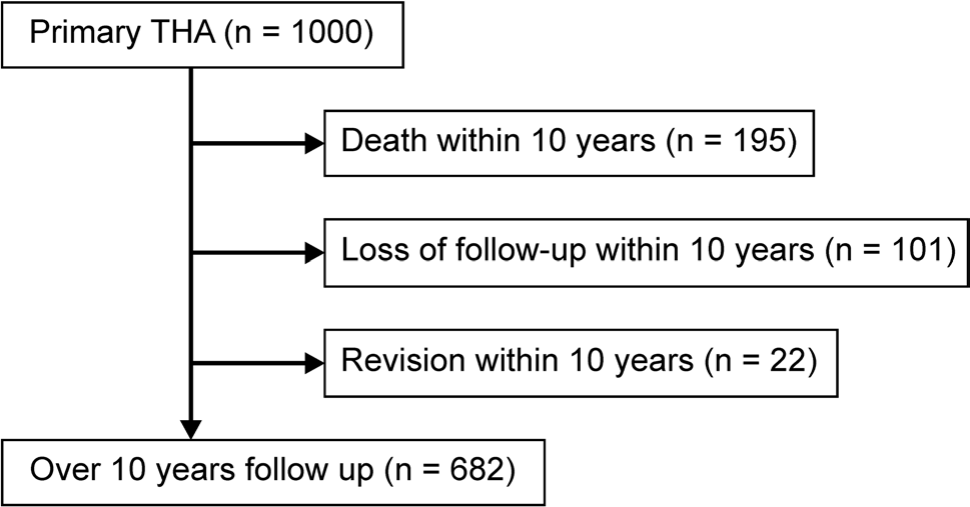
Among the 1000 primary THAs performed, 195 patients died, 101 were not available for follow-up, and 22 underwent stem revision. In total, this study included 682 patients over a minimum follow-up period of 10 years

This retrospective and descriptive multi-center follow-up study examined 1,000 primary THAs in 881 patients (mean age at the time of surgery, 62.3 [range, 23–89] years; sex, 759 [86.2%] female and 122 [13.8%] male) performed by four different surgeons at four teaching hospitals between October 2000 and December 2007. All cases operated on during this period were included, and no cases were excluded. Of these, 22 (22 hips, 2.2%) underwent revision for specific reasons, 181 (195 hips, 19.5%) died, and 86 (101 hips, 10.1%) were unavailable for follow-up (Fig. 1). In total, 597 patients (682 hips, 68.2%) were available, with a mean follow-up period of 13.3 (range, 10–18.3) years. A total of 682 hips were operated on (mean age at the time of surgery, 60.6 [range, 23–89] years; sex, 600 [88.0%] female and 82 [12.0%] male). The diagnoses included osteoarthrosis (599), idiopathic avascular necrosis (47), rheumatoid arthritis (26), previous fracture (4), ankylosing spondylitis (2), juvenile rheumatoid arthritis (2), sarcoidosis (1), and pigmented villonodular synovitis (1). The final follow-up examination was conducted in December 2018.

### Surgical technique

The posterior and lateral approaches were used in 878 and 122 hips, respectively, according to the modified Dall technique [3, 4], at the surgeon’s discretion. On the acetabular side, cementless and cement cups were used in 613 and 387 cases, respectively. On the femoral side, the Endurance bone cement (DePuy CMW, Blackpool, UK) and Surgical Simplex P radiopaque bone cement (Stryker Orthopedics, Mahwah, NJ, USA) were used in 44 and 956 cases, respectively. A stem developed for Asian populations (with a 30-mm, 33-mm, and 35.5-mm offset) was used in 280 hips (28.0%). Each surgeon selected the appropriate stem size and offset according to the patient’s physique. All surgeons used modern cementing techniques. However, one surgeon combined the interface bioactive bone–cement technique (IBBC) [5–7].

### Clinical assessment

We recorded the postoperative complications, such as dislocation, infection, deep vein thrombosis, nerve palsy, and fractures; associated factors; and surgical techniques used in THA revisions.

### Radiological assessment

For radiographic evaluation, preoperative bilateral anteroposterior (AP) images were obtained in all patients who underwent follow-up examinations. Postoperative radiolucent lines (RLLs) at the bone–cement interface were assessed using the Barack classification. At the final examination, the quality of cementation was evaluated in the radiographic images based on the Gruen zone classification. Nonlinear localized balloon-like transparent image findings were carefully evaluated for osteolysis. Radiographic loosening was assessed using the Harris classification system. Stem slip was evaluated using the Fowler method. Cortical hypertrophy (CH) was evaluated using a modified Gruen classification with zone 4 divided into medial (4 M) and lateral (4L) to analyze stress distribution [2]. Varus/valgus insertion angle (≥ 3° from neutral) was measured in postoperative radiographic bilateral AP images. All measurements were corrected for magnification using known dimensions of the femoral head. Radiographs were evaluated independently by four co-authors blinded to the patient's clinical status. Agreement was reached through consensus-based discussion in cases of discrepancies among the observers.

### Statistical analysis

Primary endpoints were any revision and stem revision for any reason at 10 and 15 years. The chi-square test was used to determine the relationship between CH and the varus/valgus insertion angle, as well as with stem offset size. Survival curves were estimated using the Kaplan–Meier method [8]. EZR (Saitama Medical Center, Jichi Medical University, Saitama, Japan) [9], a graphical user interface for R 4.3.1 (R Foundation for Statistical Computing, Vienna, Austria), was used for all statistical analyses. The significance level was set at 5%.

## Results

### Clinical assessment

Postoperative complications were as follows: dislocation (total *n* = 36; conservative, *n* = 25; revision for recurrent dislocation, *n* = 11), infection (*n* = 9, remission achieved after conservative treatment in one patient only), deep vein thromboses (*n* = 5), incomplete sciatic nerve palsy (*n* = 2, resolved with conservative treatment), and trauma-related femoral fractures (*n* = 1). Fractures were classified as Vancouver type B1, osteosynthesis combined with allogeneic bone grafting was performed, and implant revision was not required. Notably, no fractures occurred during the intraoperative period. No fatal pulmonary thromboembolisms were observed.

At the final follow-up examination, 22 hips had been revised for several reasons (Table 1). Of these, eight hips required revision only on the acetabular side, whereas 14 hips also required removal and revision of the femoral stem. Femoral revision techniques included cement-in-cement (*n* = 7) [10], in-cement (*n* = 2) [10], impaction bone grafting (*n* = 2) [11, 12], IBBC (*n* = 1), combined strut allograft and cement-in-cement (*n* = 1), and cement long stem (*n* = 1). The cement-in-cement method was used for all recurrent dislocations. The standard size Exeter stem was used for revision in 13 hips, except for one case, which was revised with a long stem.

**Table 1:** The causes of total hip arthroplasty revision (*n* = 22)

Cause of revision	Revision for any reason	Revision on the acetabular side only	Revision on the femoral side also
Dislocation	11	7	4
Infection	8	0	8
Initial fixation failure of cementless cup	2	1	1
Focal osteolysis	1	0	1
Total	22	8	14

### Radiological assessment

Among the 682 hips followed up for ≥ 10 years, 215 (31.5%) used the Asian-sized Exeter stem. The stem insertion angles were varus, valgus, and neutral in 13 (1.9%), 21 (3.1%), and 648 hips (95.6%), respectively. No RLL was observed at the bone–cement interface. Focal osteolysis was observed in 10 hips (1.5%).

Stem slip < 2 mm occurred in 88.6% of hips (Table 2). Stem slip ≥ 2 mm occurred in 67 hips, with a mean of 2.7 mm. Excessive slip (≥ 5 mm) occurred in one case due to cement fracture, with no revision required (17 mm).

**Table 2:** Stem slipping distances at the final follow-up evaluation

	*n*	%
< 1 mm	256	37.6
≥ 1 mm, < 2 mm	348	51.0
≥ 2 mm	67	9.8
Unmeasurable	11	1.6

CH was observed in 200 hips (29.3%), with 170 additional cases (24.9%) since the previous follow-up study of the present series [2]. However, CH was often identified across multiple adjacent zones: *n* = 0, zones 1 and 7; *n* = 10, zone 2; *n* = 39, zone 3; *n* = 29, zone 4L; *n* = 79, zone 4 M; *n* = 177, zone 5; and *n* = 65, zone 6. Of the 200 cases of CH, 158 (79%) occurred at the medial sides (*n* = 1, zone 4 M; *n* = 2, zone 6; *n* = 155, multiple zones, including zone 5), 20 (10%) at the lateral sides (all multiple zones, including zone 3), and 22 (11%) on both sides. The insertion angles were neutral in 196 hips (98%), varus in two hips (1%), and valgus in two hips (1%) (Table 3). The association between the incidence of CH and varus/valgus insertion was significant (*p* < 0.05). In total, Asian-sized stems were used in 58 hips (29%) (Table 3); however, the association between CH incidence and stem offset size was not statistically significant.

**Table 3:** Relationship between stem position or offset size and cortical hypertrophy

		CH ( +)	CH (-)
Stem position*1	Varus or valgus	4	30
	Neutral	196	452
Stem offset size*2	Smaller offset size*3 (30, 33, or 35.5 mm)	58	157
	Standard offset size (> 37.5 mm)	142	325

*1: Chi-square test result was *p* < 0.05

*2: Chi-square test result was not significant

*3: Asia–Pacific stem size range

CH, Cortical hypertrophy

### Survival analysis

**Fig. 2:**
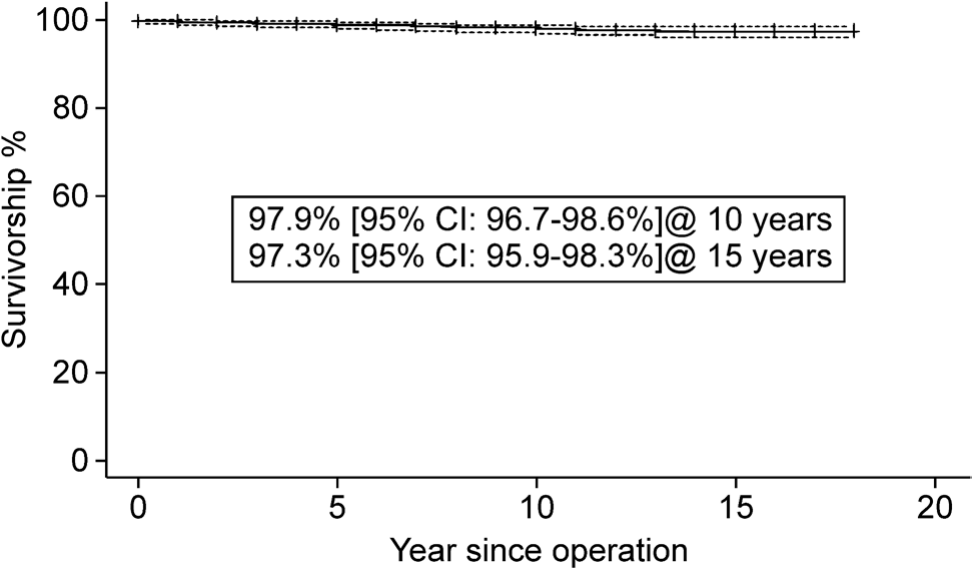
Kaplan–Meier survival analysis predicts 97.9% and 97.3% 10-year and 15-year survival, respectively, when revision for any reason is the endpoint

**Fig. 3:**
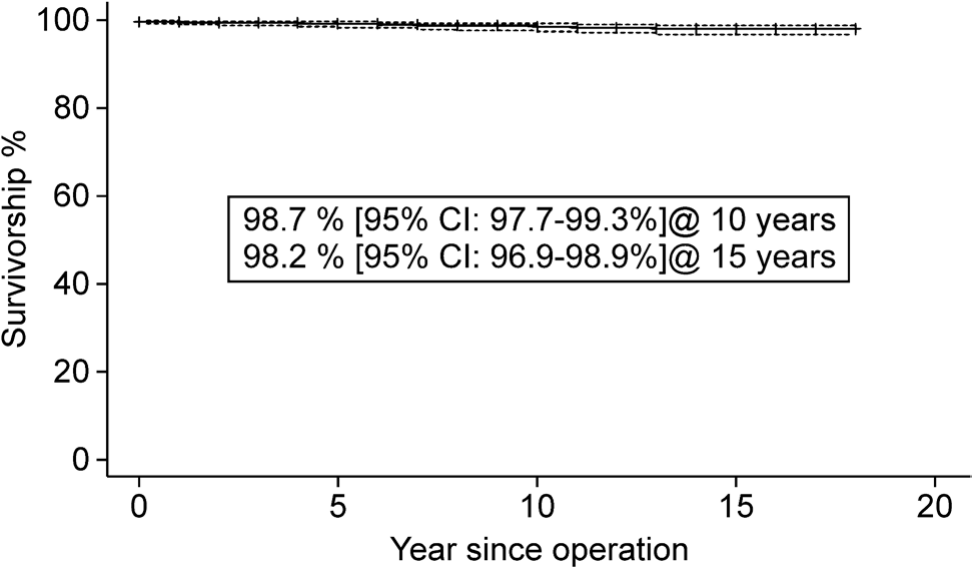
Kaplan–Meier survival analysis predicts 98.7% and 98.2% 10-year and 15-year survival, respectively, when femoral stem revision for any reason is the endpoint

Figures 2 and 3 show the Kaplan–Meier survival curves. When the endpoint was the incidence of any revision (i.e., cup only or stem included), the 10-year and 15-year survival rates were 97.9% (95% confidence interval [CI]: 96.7–98.6%) and 97.3% (95% CI: 95.9–98.3%), respectively. When the endpoint was incidence of stem revision, the 10-year and 15-year survival rates were 98.7% (95% CI: 97.7–99.3%) and 98.2% (95% CI: 96.9–98.9%), respectively.

## Discussion

In this follow-up study of 1,000 primary THAs, 14 (1.4%) required removal and revision of the femoral stem. Among the 682 primary THAs followed up for ≥ 10 years, radiographic findings indicated excellent overall results, with no cases of RLL at the bone–cement interface observed. These results were consistent with those of studies reporting the efficacy of the universal and V40 Exeter stems, even after modifications to its trunnion shape in 2001 [13, 14].

The long-term stability of the Exeter stem can be attributed to its design, which, in accordance with the tapered slip theory [1, 15], allows for controlled subsidence within the cement that primarily transmits the load from the proximal femur to the bone [16]. Typically, a 1–2-mm slip occurs in the early postoperative period and continues to progress, albeit slightly, without stopping. In this follow-up study, even after > 10 years, 88.6% of slips were < 2 mm, and only one patient exhibited an excessive slip of 17 mm due to a cement fracture. One case of excessive slip involved an inactive older adult patient with mild pain; therefore, revision was not performed. A substantial slip over time could predict the occurrence of periprosthetic or cement fracture; in cases of rapid progression or persistent pain, revision surgery may be required.

Focal osteolysis was observed in 10 hips, presenting as localized areas of transparent bone in the radiographic images. Although this phenomenon does not directly lead to stem loosening, widespread occurrence can contribute to loosening over time. Focal osteolysis may occur when the thin cement mantle ruptures and affects the bone–cement interface [17]. Therefore, the stem must be inserted with sufficient care to avoid defects to the cement mantle. In most of our cases, revision was not required, with the exception of one hip.

**Fig. 4:**
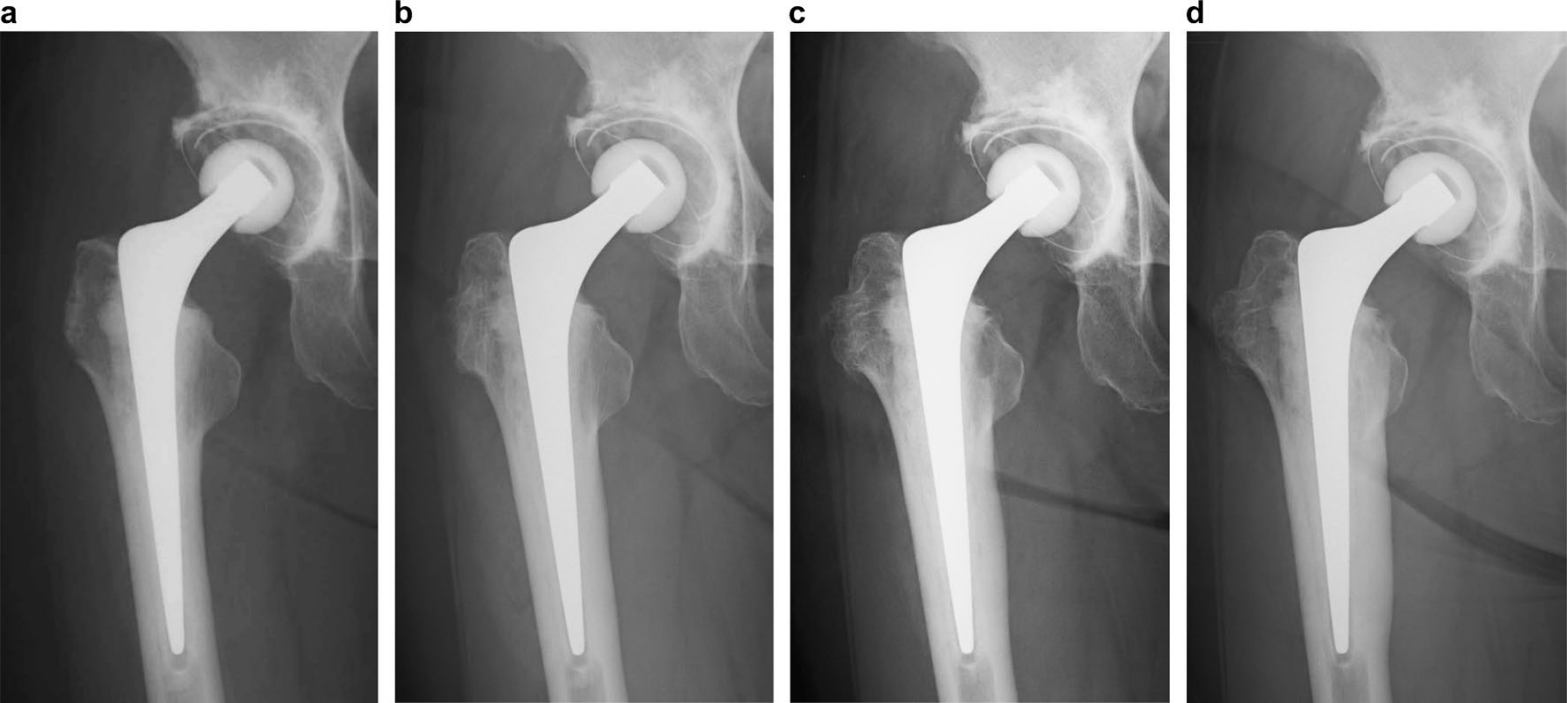
THA with grade A cementing, neutral varus/valgus insertion, and 35.5 mm offset (**a**). No CH/focal osteolysis after 4 years (**b**). CH in zones 5 and 6 after 8 years (**c**). Increased CH thickness after 11 years (**d**) (slip: 1.3 mm) CH, Cortical hypertrophy

The earliest case of CH was observed at 1 year postoperatively, with the number of cases gradually increasing thereafter. The incidence of CH increased from 92 out of 985 hips (9.5%) in the previous study by Fujita et al. to 200 out of 682 hips (29.3%) in the current study, with 170 hips (24.9%) representing new cases. While there were no cases in which the CH completely disappeared, some cases demonstrated changes in CH thickness (Fig. 4), and most of the CHs were located in zone 5. We inferred that this was the result of Exeter stem slipping within the cement mantle, which, owing to the differing taper shapes medial and lateral side, increased the load stress transmitted from the vicinity of the calcar to the distal medial portion.

Among the 200 cases showing CH, varus/valgus insertion was observed in four hips (0.5%) and Asian-sized stems were used in 67 hips (29.0%). While a significant difference was observed in the varus/valgus insertion, it resulted from a lower incidence of CH and was not the cause of CH. There was no significant difference between Asian-sized stems and CH appearance.

Fujita et al. [7] conducted a 10-year follow-up study comparing two cement stems with different designs and fixation styles. Their radiographic analysis revealed that CH was predominantly found in the lateral zones 3 and 4L of the composite beam design, and in the medial zones 4 M, 5, and 6 of the taper-slip Exeter stem design.

Although CH was observed in a large proportion of cases (29.3%) in the present study, it did not lead to loosening to the extent of periprosthetic fracture or necessitate revision. Numerous studies have explored the occurrence of CH and its impact on clinical scores and periprosthetic fractures. Iwase et al. [18], in their 10-year follow-up study of 100 hips with CH and hip pain, reported that patients with CH had better pain scores on the Japanese Orthopedic Association Hip Disease Evaluation Questionnaire [19]. In a 15-year postoperative follow-up period of THAs using cobalt chromium CPT stems (Zimmer, Warsaw, IN, USA), Baryeh et al. [20] reported CH in six out of 500 hips (1.2%) but observed no cases of periprosthetic fracture. Conversely, Oe et al. [21] used a cobalt chromium SC-stem (Kyocera, Kyoto, Japan) with a curved triple taper shape and a stainless steel C-stem (DePuy International, Leeds, UK) featuring a straight triple taper shape in a cohort of 1,777 hips. After a mean follow-up period of 5.4 (range, 3.5–6.3) years, atypical periprosthetic fractures were observed in five SC-stem hips. In four of these cases, cortical bone changes were observed in radiographic images before the fractures occurred. Similarly, Wakama et al. [22] used SC-stem in 559 hips and reported atypical periprosthetic fractures in five hips (0.9%) at 3.5–6.4 years postoperatively after local cortical bone changes occurred.

Hirata et al. [23] developed an experimental model consisting of four different metals and two types of cement with varying viscosities. Their findings demonstrated that metals with the same degree of surface roughness exhibited different friction and wettability coefficients. This suggests that the extent of femoral stem subsidence in vivo is influenced by factors such as surface roughness, metal composition, and cement viscosity. Thus, even slight variations in design shape and material properties may impact outcomes; hence, caution should be exercised.

In the present study, which involved the use of the Exeter stem in Asian patients, only one patient (0.1%) experienced a periprosthetic fracture due to trauma during the follow-up period. Palan et al. [24] compared four different brands of cemented stems for the revision of periprosthetic fractures and proposed that the rate of periprosthetic fractures varied depending on the fixation method and stem design. Kazi et al. [25] analyzed National Joint Registry data of 292,987 primary THAs performed over a follow-up period of approximately 10 years and compared the causes of revision in 5,492 hips in terms of fixation style and primary brand used, including the Exeter and Charnley stems (DePuy International). Their findings indicated that taper-slip stems were associated with a higher incidence of periprosthetic fractures, fewer cases of aseptic loosening, and lower rates of revision than those of composite beam stems. Additionally, a comparison between the Exeter stem and other tapered slip stems revealed that the Exeter stem exhibited a lower risk of periprosthetic fracture-related revision. Notably, the Exeter stem demonstrated the highest survival rate.

The present study had several limitations. First, longitudinal clinical outcomes were not included because these data were not obtained from one institution, which accounted for > 50% of the participants. Second, despite the large sample size, many patients died or dropped out, resulting in long-term follow-up being achieved in only 68.2% of participants. Third, this was an observational study that had no control series.

In conclusion, this was the first study to investigate the use of the Exeter stem in Japan with a follow-up period > 10 years and reported excellent midterm results when the Exeter stem and modern cementing techniques were used for primary THAs in Japan. Importantly, no cases of aseptic loosening were observed. To determine the performance of the Exeter stem among Asian populations, further studies from additional centers and longer follow-up periods are needed. Overall, the Exeter stem shows great promise in improving the long-term outcomes of THAs in Asian populations, particularly in cases involving patients with smaller physiques.

## Data Availability

The datasets used and/or analyzed during the current study are available from the corresponding author on reasonable request.

## References

[1] Ling RS, Charity J, Lee AJ, Whitehouse SL, Timperley AJ, Gie GA (2009) The long-term results of the original Exeter polished cemented femoral component: a follow-up report. J Arthroplasty 24:511–517. 10.1016/j.arth.2009.02.002 19282139 10.1016/j.arth.2009.02.002

[2] Fujita H, Katayama N, Iwase T, Otsuka H (2012) Multi-center study of use of the Exeter stem in Japan: evaluation of 1000 primary THA. J Orthop Sci 17:370–376. 10.1007/s00776-012-0237-5 22552547 10.1007/s00776-012-0237-5

[3] Kataoka M, Fujita H, Hara H, Harada H, Okutani Y, Murotani Y (2021) Influence of the knot position on the union of the greater trochanter after bipolar hip arthroplasty via the modified Dall approach: a prospective non-randomized study. BMC Musculoskelet Disord 22:162. 10.1186/s12891-021-04005-1 33568142 10.1186/s12891-021-04005-1 PMC7877116

[4] Hara H, Fujita H, Okutani Y, Kataoka M, Harada H, Murotani Y (2022) The influence of anterior and posterior knot placement on hip function after total hip arthroplasty using a modified Dall’s approach: a prospective non-randomised comparative study. Hip Int 32:443–451. 10.1177/1120700020977789 33297766 10.1177/1120700020977789

[5] Oonishi H, Ohashi H, Oonishi H, Kim SC (2008) THA with hydroxyapatite granules at cement-bone interface: 15- to 20-year results. Clin Orthop Relat Res 466:373–379. 10.1007/s11999-007-0057-7 18196420 10.1007/s11999-007-0057-7 PMC2505131

[6] Fujita H, Oonishi H, Ito S, Kim SC, Doukawa H (2008) Radiological evaluation of the femoral component fixed with interface bioactive bone cement in revision total hip arthroplasty. J Arthroplasty 23:689–693. 10.1016/j.arth.2007.05.042 18534378 10.1016/j.arth.2007.05.042

[7] Fujita H, Hara H, Harada H, Kataoka M, Tominaga T, Nishimura R (2020) Prospective, comparative study of cemented, smooth-surfaced titanium stems and polish-surfaced, stainless steel stems at a minimum follow-up of 10 years. Eur J Orthop Surg Traumatol 30:501–512. 10.1007/s00590-019-02597-y 31741055 10.1007/s00590-019-02597-y

[8] Kaplan EL, Meier P (1958) Nonparametric estimation from incomplete observations. J Am Stat Assoc 53:457–481. 10.1080/01621459.1958.10501452 10.1080/01621459.1958.10501452

[9] Kanda Y (2013) Investigation of the freely available easy-to-use software “EZR” for medical statistics. Bone Marrow Transplant 48:452–458. 10.1038/bmt.2012.244 23208313 10.1038/bmt.2012.244 PMC3590441

[10] Fujita H, Katayama N, Iwase T, Otsuka H (2022) Multi-centre study of cement-in-cement and in-cement femoral revision total hip arthroplasty using polished, stainless steel stems. J Orthop Sci 27:1073–1077. 10.1016/j.jos.2021.06.017 34391617 10.1016/j.jos.2021.06.017

[11] Iwase T, Otsuka H, Katayama N, Fujita H (2012) Impaction bone grafting for femoral revision hip arthroplasty with Exeter Universal stem in Japan. Arch Orthop Trauma Surg 132:1487–1494. 10.1007/s00402-012-1561-0 22684741 10.1007/s00402-012-1561-0

[12] Iwase T, Otsuka H, Katayama N, Fujita H (2024) Impaction bone grafting for femoral revision hip arthroplasty with Exeter stem in Japan: an extended 10- to 15-year stem survival analysis of the previously reported series. J Orthop Sci 29:151–156. 10.1016/j.jos.2022.12.009 36610839 10.1016/j.jos.2022.12.009

[13] Carrington NC, Sierra RJ, Gie GA, Hubble MJ, Timperley AJ, Howell JR (2009) The Exeter Universal cemented femoral component at 15 to 17 years: an update on the first 325 hips. J Bone Joint Surg Br 91:730–737. 10.1302/0301-620X.91B6.21627 19483224 10.1302/0301-620X.91B6.21627

[14] Westerman RW, Whitehouse SL, Hubble MJ, Timperley AJ, Howell JR, Wilson MJ (2018) The Exeter V40 cemented femoral component at a minimum 10-year follow-up: the first 540 cases. Bone Joint J 100(8):1002–1009. 10.1302/0301-620X.100B8.BJJ-2017-1535.R1 30062940 10.1302/0301-620X.100B8.BJJ-2017-1535.R1

[15] Shen G (1998) Femoral stem fixation. an engineering interpretation of the long-term outcome of Charnley and Exeter stems. J Bone Joint Surg Br 80:754–756. 10.1302/0301-620x.80b5.8621 9768879 10.1302/0301-620x.80b5.8621

[16] Iwase T, Morita D, Takemoto G, Fujita H, Katayama N, Otsuka H (2019) Peri-prosthetic bone remodeling and change in bone mineral density in the femur after cemented polished tapered stem implantation. Eur J Orthop Surg Traumatol 29:1061–1067. 10.1007/s00590-019-02414-6 30848380 10.1007/s00590-019-02414-6

[17] Kawate K, Ohmura T, Hiyoshi N, Natsume Y, Teranishi T, Tamai S (1999) Thin cement mantle and osteolysis with a precoated stem. Clin Orthop Relat Res 365:124–129. 10.1097/00003086-199908000-00017 10.1097/00003086-199908000-00017 10627696

[18] Iwase T, Morita D, Takemoto G (2020) The effects of patient characteristics and stem alignment on distal femoral cortical hypertrophy after cemented polished tapered stem implantation. Eur J Orthop Surg Traumatol 30:559–567. 10.1007/s00590-019-02605-1 31853636 10.1007/s00590-019-02605-1

[19] Matsumoto T, Kaneuji A, Hiejima Y, Sugiyama H, Akiyama H, Atsumi T et al (2012) Japanese orthopaedic association hip disease evaluation questionnaire (JHEQ): a patient-based evaluation tool for hip-joint disease. The subcommittee on hip disease evaluation of the clinical outcome committee of the Japanese orthopaedic association. J Orthop Sci 17:25–38. 10.1007/s00776-011-0166-8 22045450 10.1007/s00776-011-0166-8 PMC3265722

[20] Baryeh K, Wang C, Sochart DH (2023) Periprosthetic femoral fractures around the original cemented polished triple-tapered C-stem femoral implant: a consecutive series of 500 primary total hip arthroplasties with an average follow-up of 15 years. Arch Orthop Trauma Surg 143:4511–4518. 10.1007/s00402-022-04712-x 36447057 10.1007/s00402-022-04712-x PMC9708125

[21] Oe K, Iida H, Hirata M, Kawamura H, Ueda N, Nakamura T, Okamoto N, Saito T (2023) An atypical periprosthetic fracture in collarless, polished, tapered, cemented stems of total hip arthroplasty: a report of five SC-stem cases and literature review. J Orthop Sci 28:1422–1429. 10.1016/j.jos.2021.04.003 34045138 10.1016/j.jos.2021.04.003

[22] Wakama H, Okamoto Y, Okayoshi T, Ikeda K, Matsuyama J, Otsuki S, Neo M (2024) Unfavorable cortical hypertrophy potentially predisposes to periprosthetic “axe splitter” fracture in a collarless polished curved triple-tapered cemented stem: the time-dependent radiographic change in five SC-stem cases. J Orthop Sci 29:439–444. 10.1016/j.jos.2022.09.004 36182639 10.1016/j.jos.2022.09.004

[23] Hirata M, Oe K, Kaneuji A, Uozu R, Shintani K, Saito T (2021) Relationship between the surface roughness of material and bone cement: an increased “polished” stem may result in the excessive taper-slip. Materials (Basel) 14:3702. 10.3390/ma14133702 34279273 10.3390/ma14133702 PMC8269856

[24] Palan J, Smith MC, Gregg P, Mellon S, Kulkarni A, Tucker K, Blom AW, Murray DW, Pandit H (2016) The influence of cemented femoral stem choice on the incidence of revision for periprosthetic fracture after primary total hip arthroplasty: an analysis of national joint registry data. Bone Joint J 98(10):1347–1354. 10.1302/0301-620X.98B10.36534 27694588 10.1302/0301-620X.98B10.36534

[25] Kazi HA, Whitehouse SL, Howell JR, Timperley AJ (2019) Not all cemented hips are the same: a register-based (NJR) comparison of taper-slip and composite beam femoral stems. Acta Orthop 90:214–219. 10.1080/17453674.2019.1582680 30838914 10.1080/17453674.2019.1582680 PMC6534220

